# Efficacy of platelet-rich plasma applied to post-extraction retained lower third molar alveoli. A systematic review

**DOI:** 10.4317/medoral.19444

**Published:** 2013-12-07

**Authors:** Cristina Barona-Dorado, Iria González-Regueiro, María Martín-Ares, Oscar Arias-Irimia, José M. Martínez-González

**Affiliations:** 1 Associate lecturer in buccal surgery. Complutense University of Madrid, Spain; 2 Resident on Master’s program in oral surgery and implant dentistry, Virgen de La Paloma Hospital, Madrid, Spain; 3 Dental doctor. Master of oral surgery and implant dentistry. Complutense University of Madrid, Spain; 4 Lecturer on the Master’s program in oral surgery and implant dentistry, Virgen de La Paloma Hospital, Madrid, Spain; 5 Professor of maxillofacial surgery. Complutense University of Madrid, Spain

## Abstract

Dental retentions have a high prevalence among the general population and their removal can involve multiple complications. The use of platelet rich plasma has been proposed in an attempt to avoid these complications, as it contains high growth factors and stimulates diverse biological functions that facilitate the healing of soft and hard tissues. Objectives: To evaluate the available scientific evidence related to the application of platelet-rich plasma in the post-extraction alveoli of a retained lower third molars. Material and Methods: A systematic review of published literature registered in the Medline, EMBASE, Cochrane and NIH databases. The following categories were included: human randomized clinical studies. Key search words were: platelet rich plasma; platelet rich plasma and oral surgery; platelet rich in growth factors and third molar. Results: Of 101 potentially valid articles, seven were selected, of which four were rejected as they failed to meet quality criteria. Three studies fulfilled all selection and quality criteria: Ogundipe et al.; Rutkowski et al.; Haraji et al. The studies all measured osteoblast activity by means of sintigraphy, and also registered pain, bleeding, inflammation, temperature, numbness as perceived by the patients, radiological bone density and the incidence of alveolar osteitis. Conclusions: Scientific evidence for the use of PRP in retained third molar surgery is poor. For this reason randomized clinical trials are needed before recommendations for the clinical application of PRP can be made.

** Key words:**Platelet rich plasma, lower third molar surgery, postoperative.

## Introduction

The use of platelet-rich plasma (PRP) is one recently proposed approach to managing complications in retained/impacted lower third molar surgery. Various authors have described PRP as an effective means for improving the healing of both hard and soft tissues, resulting in reductions in pain, inflammation and trismus, as well as an acceleration of the bone regeneration process. However, there is some controversy in the literature, which might be due to differing protocols for obtaining PRP (centrifugation) and the low numbers of systematic studies carried out to date (1,2).

PRP contains high concentrations of growth factors that stimulate different biological functions such as chemotaxis, angiogenesis, cell proliferation and differentiation, all of which facilitate healing (1-3); so when the platelets release growth factors, they trigger a process of tissue regeneration. In addition to growth factors, granulation tissue in wounds treated with PRP contains intra- and extra-platelet components that could also contribute to tissue regeneration (4,5).

PRP presents a low risk of infection or immunological reactions, as the platelets play an important role in host defense mechanisms at the wound site, due to a signal peptide release that attracts macrophages. The antimicrobial activity of platelet concentrates on the various bacteria species involved in oral infection has also been cited (1-5).

Recently, several randomized clinical trials (RCT) have been performed with small sample sizes and short follow-up periods, which have shown the results of PRP application to third molar surgical sites in the short term (6-12). The aim of the present study was to evaluate the scientific evidence in support of PRP application to retained lower third molar post-extraction alveoli.

## Material and Methods

*Bibliography Search 

A search was made for articles on platelet-rich plasma among the published biomedical literature included in the following data-bases: Medline (via Pubmed); EMBASE (via Ovid); NIH; and the Cochrane Central Register of Controlled Trials. The search was extended to include systematic literature reviews in the Medline database and the Cochrane Database of Systematic Reviews. The search was limited to randomized clinical studies of human, regardless of study duration or the language of publication.

*Search Strategy

The search covered all literature published up to and including June 30th 2013. The search parameters were “randomized clinical trials on humans”. The key search words used were.

- In the Medline database: platelet-rich plasma and oral surgery; plasma rich in growth factors and third molar.

- In the EMBASE database: platelet rich plasma and oral surgery.

- In the Cochrane database: platelet rich plasma.

- In the NIH database: platelet rich plasma and oral surgery.

*Article Selection Criteria

-Inclusion criteria

Studies with the following characteristics were included:

Population: studies of humans that included adult patients who had undergone extractions of one or more retained lower third molars.

Intervention: application of platelet-rich plasma to post-extraction lower third molar alveoli.

Comparison: application of a placebo to post-extraction retained lower third molar.

Result variables assessed: pain, inflammation, bleeding, degree of healing, incidence of alveolar osteitis, degree of radiological bone regeneration and osteoblast activity.

Study design: only randomized clinical trials (RCTs) were included of parallel groups or split-mouth, one group having received an application of PRP and the other a placebo, with the two applications distributed randomly.

-Exclusion criteria

- Articles dealing with platelet-rich plasma applications to extraction sites of teeth other than retained lower third molars.

- Randomized clinical trials combining platelet-rich plasma application with other bone regeneration materials.

- Articles duplicating the same trial or study population as another, obtaining the same results, but using different study periods, or published in more than one journal.

*Data extraction 

Relevant data were extracted from the studies that met the inclusion criteria and collated in tables of scientific evidence that registered the following data.

- Authors and year of publication.

- Study design: clinical trial of parallel groups or split mouth.

- Patient selection criteria.

- Sample size.

- Protocol for obtaining PRP.

- Initial patient characteristics.

- Result variables.

Quality evaluation and synthesis of scientific evidence 

A critical assessment of the selected studies was performed, evaluating their internal and external validity. This was done by means of the Jadad scale (13), which awards a score of zero to five according to whether or not the following criteria were met:

- Whether or not the study was randomized.

- Whether or not it was double blind.

- Whether or not it listed patients lost or retired from the study procedure.

- Whether or not the method for generating the randomization sequence (if described) was adequate.

According to the Jadad scale, a clinical trial is considered of poor quality if it scores less than three. On the basis of the information extracted and collated in tables of scientific evidence and having assessed the quality of the clinical trials, the evidence identified as being of adequate quality was then organized, synthesized, and structured.

*Classification of scientific evidence 

The quality of scientific evidence was classified by means of the GRADE system (14).

## Results

1- Search Results. Flow diagram

a) Systematic reviews and meta-analyses

Five systematic reviews were found (1,2,5,15,16), all of which were excluded because: one (1) included clinical trials that had combined PRP with other bone regeneration materials, and had applied these to not only retained lower third molar post-extraction alveoli; another review (15) included clinical studies in which bone defects were treated by means of periodontal defect regeneration or maxillary sinus elevation; two (2,16) analyzed the characteristics of platelet-rich plasma, techniques for obtaining it and its possible applications, rather than treating lower third molar sites; the last (5) did not fall within the field of oral surgery but applied PRP to chronic cutaneous wounds.

b) Randomized clinical trials 

The search identified 101 articles, of which 16 were duplications. Of the remaining 85, 72 were discarded on the basis of the title, as it was clear that they did not correspond to the application of platelet rich plasma in retained lower third molar surgery.

In this way, 13 articles were identified that were potentially adequate for inclusion. Having reviewed the abstracts, seven articles that met all the proposed inclusion criteria were selected and their complete texts were read; all were randomized clinical trials (6-12).

**Figure 1:**
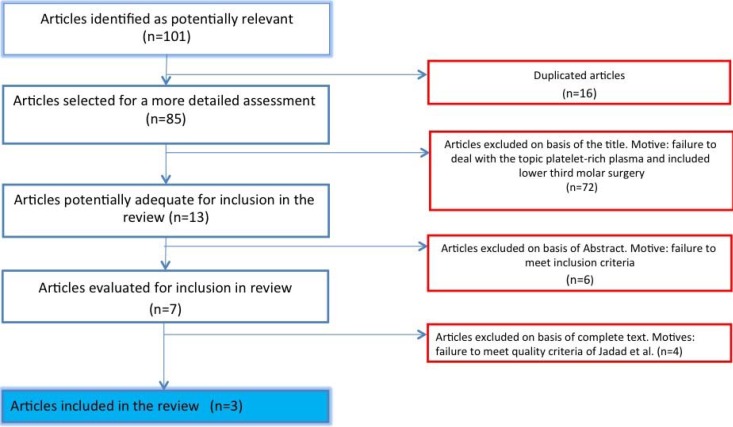
Flow diagram of randomized clinical trial selection process.

After evaluating the quality and potential for bias by means of the Jadad scale (13), four were eliminated as they failed to achieve the minimum score required (6-9) ( Table 1). Figure 1 is a flow diagram describing the RCT selection process.

**Table 1:**
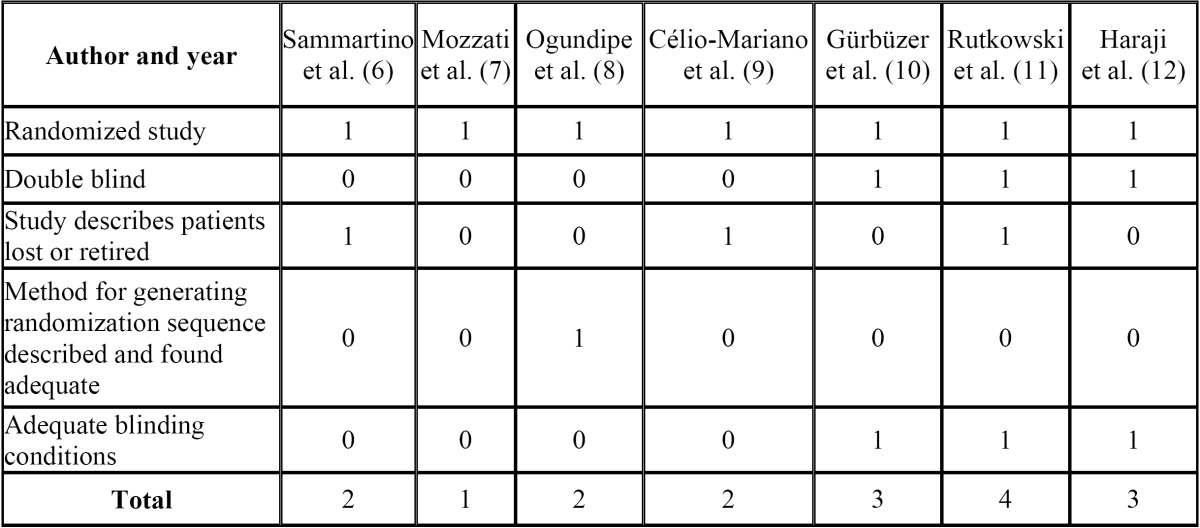
Results of quality evaluation generated by the Jadad scale (13) for RCTs initially selected.

2- Qualitative Synthesis 

The results extracted from the RCTs selected are shown in  Table 2.

**Table 2:**
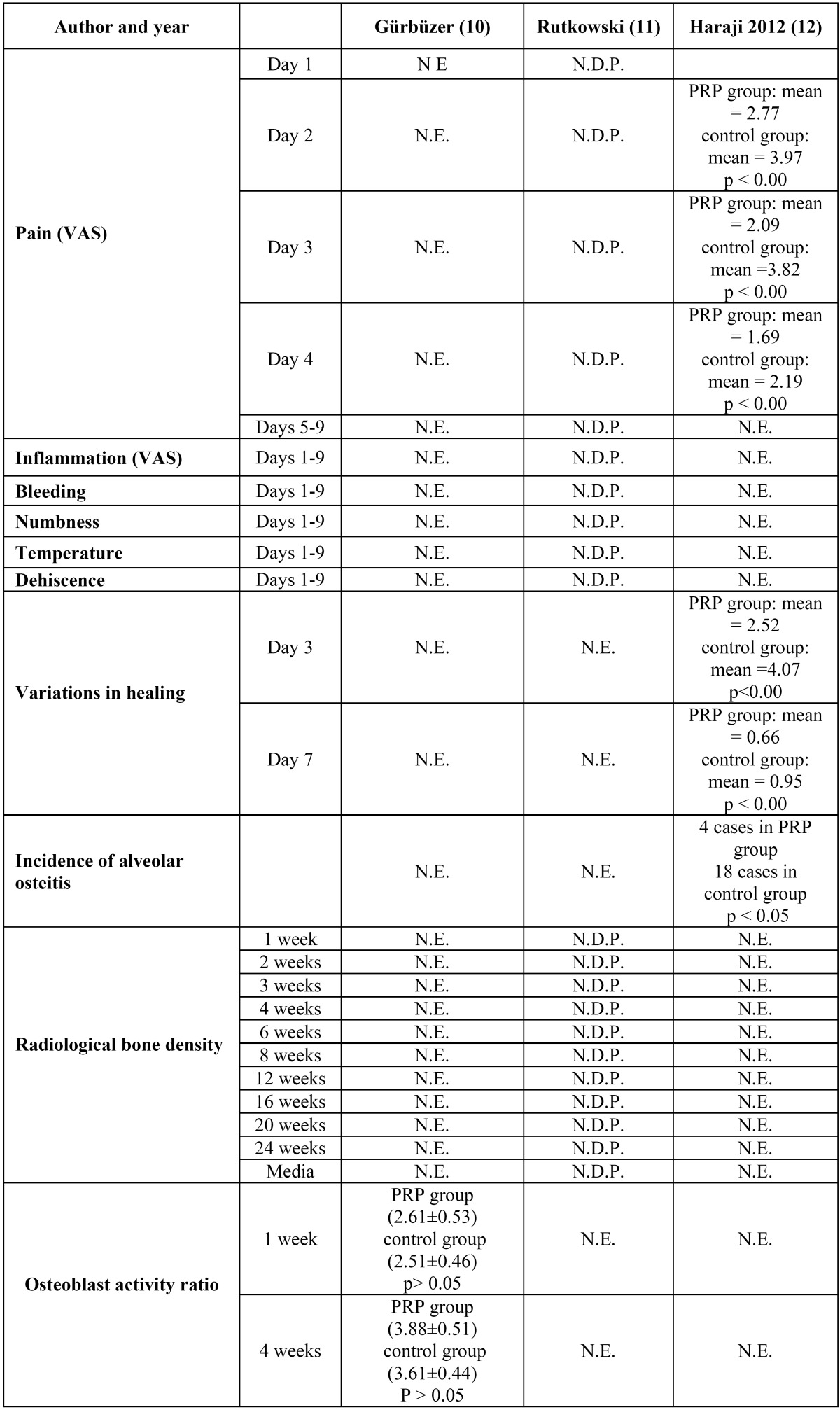
Results of studies included for analysis. (VAS = visual analogue scale; N.E. = not evaluated; N.D.P. = no data provided; PRP = platelet-rich plasma).

Gürbüzer et al. (10) is a split-mouth study of 12 patients with bilateral retained lower third molars. The study objective was to investigate the short-term effects of PRP on osteoblast activity during the alveolar healing process after retained mandibular third molar extraction. Osteoblast activity was measured in alveolar neoformed bone by means of scintigraphy, one and four weeks after surgery.

16 ml of blood were extracted in two 8.5 ml tubes with citrate phosphate dextrose as anticoagulant; double-centrifugation was performed, with an initial ten-minute centrifugation at 2400 rpm and a final centrifugation at 3600 rpm for 15 minutes. The PRP obtained was diluted in saline solution at a concentration of 1:5. Lastly, the patients own blood and 0.5 ml of calcium chloride were added.

Bone scintigraphy with technetium-99 was performed one and four weeks after surgery. No statistically significant differences were found between groups.

Rutkowski et al. (11) carried out a split-mouth study of six patients. Only non-smokers took part, who had bilateral retained lower third molars in similar states of eruption and without medical antecedents.

Two tubes of 4.5 ml of blood were obtained with sodium citrate as anticoagulant. These were centrifuged for 10 minutes at 1150 g. The plasma was aspirated with a pipette, 3mm above and 2 mm below the interface between plasma and the red blood cell layer. Extraction of the lower third molars was performed, after which Gelfoam® was applied to a control group and Gelfoam® together with PRP to the study group.

The study measured pain, inflammation, bleeding and temperature perceived by the patient, using a visual analogue scale nine days after surgery. No statistically significant differences were observed between the PRP group and the control group for any of the parameters analyzed.

The study also evaluated dehiscence, bleeding, inflammation and intraoral swelling perceived by an observer. A significant difference was observed in favor of PRP in relation to facial swelling.

Digital periapical radiographs were made using VinWix Pro® software to determine bone density by means of gray scales. The mean radiological bone density was significantly greater in the group treated with PRP than in the control group (Student t test for paired samples: p<0.05).

Haraji et al. (12) set out to evaluate the prophylactic effect of PRP against alveolar osteitis, as well as its effect on pain management and the acceleration of the healing process. Forty patients were selected with bilateral maxillary or mandibular third molars that presented similar levels of surgical difficulty and similar risk factors for alveolar osteitis: antecedents of pericoronaritis, treatment with oral contraceptives, smoking, bruxism or antecedents of dry alveolitis.

10 ml. of blood were extracted in 1 ml tubes with sodium citrate at 3.8% as anticoagulant and centrifuged at 460 g for 8 minutes. The portion of platelet-rich plasma was separated from the red blood and calcium chloride was added (0.05 ml. per ml of plasma). It was placed in an oven and heated at 37º for 5-8 minutes.

After surgery, pain was evaluated by means of a visual analogue scale; healing was evaluated by means of observation of coagulate degeneration, wound dehiscence with suppuration, wound dehiscence without suppuration (or non-healing). It was found that the intensity of post-operative pain was significantly less (p<0.00) and healing better (p<0.00) in the PRP group than in the control group. Incidence of post-operative alveolar osteitis on the side treated with PRP was significantly less (p<0.05) than on the conlateral side.

## Discussion

Platelet-rich plasma is used in a variety of clinical situations in the field of oral surgery, ranging from filling post-extraction alveoli to more complex surgery involving bone regeneration or sinus elevation (17-19). It is claimed that its use reduces pain and inflammation, accelerates the epithelialization of soft tissues and promotes bone regeneration (20-21). The objective of this syste-matic literature review was to analyze the scientific evidence available for PRP application in retained lower third molar surgery.

Although there are many authors who extol the virtues of PRP use, there are few randomized clinical trials that have studied this topic. The present review could only find seven clinical trials on the subject, of which four failed to meet Jadad criteria (13) for avoiding bias.

The three remaining studies included for review - Gürbüzer et al. (10), Rutkowski et al. (11), and Haraji et al. (12) - did not present any bias in relation to the clinical trial procedure, but two did present problems when it came to communicating the results.

Haraji et al. (12) affirm that pain and inflammation levels in the study group treated with PRP were lower than in the control group with statistically significant difference (p<0.00). However, the article did not include any values (mean, standard deviation or any numeric value) deriving from the trial. This article also determined the incidence of osteitis but failed to specify the method employed for its diagnosis. Furthermore, 80 third molar extractions were performed – 40 maxillary and 40 mandibular – all of which were included in the results. This represents an important fault in methodology, given that, from a clinical point of view, the post-operative conditions of a lower third molar cannot be compared with those of an upper third molar.

Similarly, Rutkowski et al. (11) state that there were no differences between groups for the incidence of pain but a statistically significant reduction in inflammation in the PRP group. They found differences in the degree of ossification using digital radio-graphy in favor of PRP. However, they fail to provide objective data for any of the variables studied and limit their results to the statistical analysis applied to generate p-values. In addition, the patient sample was small, only six patients and twelve lower third molars. As these two published studies failed to provide any data on pain or inflammation, and furthermore, their sample sizes were small, it is impossible to carry a synthesis of these results by means of meta-analysis.

The third clinical trial included for analysis, published by Gürbüzer et al. (13), measured osteoblast activity one week and four weeks after surgery. The study does communicate the results clearly but fails to find statistically significant differences between groups. No other study of this type can be found that would allow a comparison with these results.

Another feature of the trials was the method employed for obtaining the platelet concentrate. None of the studies analyzed used the same method, a key factor given that the concentration of growth factors will differ depending on the method used. It is even possible that some of the less rigorous protocols may have allowed the inclusion of red cells or white cells, which would produce a stronger inflammatory response than expected (11).

Recently, the Spanish Agency of Medicines and Medical Products has published a report that considers PRP application a medication fit for human use. Authorization of any medication implies that it meets the required criteria of quality, safety and efficacy. However, the report states that clinical trials of sufficient quality are yet to be carried out before firm conclusions can be drawn as to its application and urges researchers to carry out clinical trials of adequate design in order to establish an adequate body of evidence for each pathology, type of PRP and application (Alonso C, Baró F, Blanquer M, de Felipe P, Fernández ME, Gómez-Chacón C, et al. INFORME/ V1/23052013, Spanish Agency of Medicines and Medical Products on the use of plate-let-rich plasma, Ministry of Health, Social Services and Equality, available at: http://www.aemps.gob.es/vigilancia/medicamentosUsoHumano/docs/notificacion-SRA.pdf.).

When the Grade system guidelines were applied (14), the quality of published evidence for PRP application in lower third molar post-extraction alveoli was found to be poor and so recommendations for its clinical use cannot be made.

Clearly, randomized clinical studies are needed that investigate the safety and efficacy of PRP post-operative treatments for lower third molar alveoli and compare PRP applications with a placebo. Result variables should include its influence on pain, inflammation and trismus, bone and soft tissue healing and reduction of the periodontal sac distal of the adjacent second molar. Publication of the results of such trials should follow the Consort Declaration guidelines (22) in order to ensure the external validity of the results obtained.
